# Near infrared photothermoelectric effect in transparent AZO/ITO/Ag/ITO thin films

**DOI:** 10.1038/s41598-021-03766-y

**Published:** 2021-12-21

**Authors:** C. Bianchi, A. C. Marques, R. C. da Silva, T. Calmeiro, I. Ferreira

**Affiliations:** 1 CENIMAT/I3N, Department of Materials Science, NOVA School of Science and Technology, Largo da Torre, 2829-516 Caparica, Portugal; 2 IPFN-IST/UL, Instituto de Plasmas E Fusão Nuclear, Instituto Superior Técnico, Universidade de Lisboa, Estrada Nacional 10, 2695-066 Bobadela, Portugal

**Keywords:** Energy science and technology, Engineering, Optical materials and structures, Materials for optics, Thermoelectrics

## Abstract

A new concept of oxide-metal-oxide structures that combine photothermoelectric effect with high reflectance (~ 80%) at wavelengths in the infrared (> 1100 nm) and high transmittance in the visible range is reported here. This was observed in optimized ITO/Ag/ITO structure, 20 nm of Silver (Ag) and 40 nm of Indium Tin Oxide (ITO), deposited on Aluminum doped Zinc Oxide (AZO) thin film. These layers show high energy saving efficiency by keeping the temperature constant inside a glazed compartment under solar radiation, but additionally they also show a photothermoelectric effect. Under uniform heating of the sample a thermoelectric effect is observed (S = 40 mV/K), but when irradiated, a potential proportional to the intensity of the radiation is also observed. Therefore, in addition to thermal control in windows, these low emission coatings can be applied as transparent photothermoelectric devices.

## Introduction

Metal oxides are generally broadband (~ 3 eV) materials transparent to the visible range of the electromagnetic radiation spectrum and electrical insulators. A small concentration of doping elements introduced in the crystalline structure induces non-stoichiometry and a controllable number of vacancies responsible for a huge increase in electrical conductivity and a multifunctionality that allows their use in several different applications. Thin film solar cell electrodes^1^, light emitting diodes (LEDs)^2^, transparent antennas^3^, transparent supercapacitors^4–6^, triboelectric nanogenerators^7^, or photonic artificial synapses^8^ are some laboratory and commercial examples where transparent and conductive oxides (TCOs) have been applied. Their absorption in the ultraviolet (UV) wavelength range and electron generation (photosensitivity to UV) has been explored in applications such as ozone sensors^9^, UV sensors^10^, photomemory devices^11^ and in those based in the photothermoelectric effect^12^. The control of vacancies, i.e. of dopant concentration in transparent oxides, enables obtaining transparent semiconductor oxides (TSOs). TSOs have boosted new concepts of transparent transistors^13^, non-volatile memory^14^ and thermoelectric devices^15^. Despite the wide variety of oxides, the most commonly used in optoelectronics as TCOs are ITO (In_2_O_3_:Sn), AZO (ZnO:Al) and FTO (SnO_2_:F)^16^. The photothermoelectric effect in AZO^17^ and ZnO^12^ under UV light is due to the excitation of charges from the valence band to the conduction band and consequent variation in material resistance^14^. In addition to the optoelectronic properties, the optical characteristics of TCOs have also allowed their applications in low emissivity (*low-e*) glass window coatings. The emissivity of a material is the effectiveness in emitting energy as thermal radiation, compared to that of a blackbody. A blackbody has an emissivity of 100% while for a perfect reflector it is zero^18^. This is an important application as the heat losses in buildings are mainly related to heat escaping/entering through the glass windows^19^. The energy consumption of buildings represent about 40% of the total energy use in Europe and is mainly consumed to heating, cooling and lighting^20^. *Low-e* coating glass windows^21^ are a requirement to achieve the European Union’s objectives of reducing the consumption of buildings by 50% by 2050^20^. Conventional glass windows in buildings allow visibility (visible wavelength range: 380–750 nm) to the outside, but infrared radiation from the solar spectrum (> 750 nm) enters the building and causes warmup. Comfort is established by blocking or minimizing the infrared from outside to inside (in warm weather) or from inside to outside (in cold weather)^22^. As such, high transmittance in the visible and high reflectance in the infrared wavelength ranges are the basic requirements for an ideal *low-e* coating for glass windows^23^. TCOs have been reported as *low-e* coatings^20^, but common *low-e* coatings are oxide-metal multilayers^24^. The thickness of the metal layer controls the percentage of reflected and transmitted light and its wavelength band. In TCOs, this adjustment is made by tuning the dopant concentration, as the NIR absorbance usually increases with increasing dopant level^25^. Ag is often used as metal layer in oxide-metal-oxide multilayers, however some studies have shown several advantages such as the durability in using Cu or Al instead^26^, while TiO_2_^27^, ZnO^28^ or SnO_2_^29^ are common as oxide layers. Although ITO/Ag/ITO structures^1^ as *low-e* coatings have been little reported in the literature, electrodes in photovoltaic solar cells are their main application. In general, the electrical properties of TCO/metal/TCO layers are enhanced compared to TCOs, keeping the transmittance high^1,26,30–34^.

In this work, the metal layer thickness in the ITO/Ag/ITO (IAI) coatings was optimized to enable high reflection in the infrared and high transmittance in the visible, and a photothermoelectric effect (PTE) when deposited on AZO thin films. The PTE was first described by *Tauc* for germanium, who also developed a relationship for the change in thermoelectric voltage under weak illumination^35^. PTE effect consists of a non-uniform absorption/reflection of light in a device/material that induces a temperature difference, ∆T, driving a current and producing a voltage through the Seebeck effect. Examples of applications that can benefit from PTE effect, as a green energy conversion process are those of optical sensing and of conversion of solar energy into electricity^36^. Several materials have shown potential for photothermoelectric applications, mostly low-dimensional systems, such as carbon nanotubes^37^, nanowires^38^, graphene^39^, nanoporous silicon^40^ or single-layer MoS_2_^41^. The performance of PTE devices reported to date has been limited to the use of a focused laser spot or non-uniform illumination to achieve the photovoltage^37,41^. Here we demonstrate photothermal voltage on a multillayer structure under uniform solar radiation. Besides, converting infrared energy of solar spectrum into electricity also enables thermal control through windows. This concept is reported for the first time and is correlated with optical properties, temperature and light radiation effects.

## Methods

### Thin film deposition

**Figure 1:**
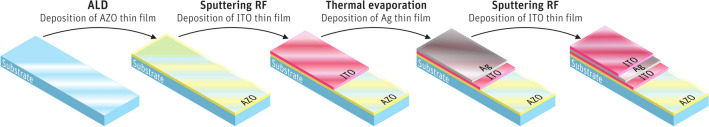
Schematic of photothermoelectric layers structure.

Indium tin oxide (ITO) layers about 40 nm thick were deposited by Radio Frequency (RF) Magnetron Sputtering (*Mantis)* on borosilicate glass (1 mm thick, as supplied by *Marienfeld),* and polyimide (Kapton HN, 50 µm thick as supplied by *DuPont*) substrates with and without AZO coatings. An In_2_O_3_:SnO_2_ (90:10 wt%) target (with purity of 99.99%, supplied by *Super Conductor Materials Inc*) was used and 1 h deposition was performed at 12.5 sccm Ar gas flow, 60 W RF power and 2 × 10^−3^ mbar pressure. The silver layers were obtained by resistive thermal evaporation (*Korvus Technology*) of Ag pellets (*Super Conductor Materials, Inc*.) with 99.99% purity in a tungsten boat. The pressure inside the thermal evaporation chamber was maintained around 7 × 10^−6^ mbar and the thickness was set to be around 10, 20 and 30 nm. The AZO thin films were deposited by Atomic Layer Deposition (ALD) on glass or polyimide substrate, according to the previous study^17^. Figure 1 shows the sequence of steps followed to produce the photothermoelectric device.

### Characterization

The film thicknesses of all samples were measured with a mechanical stylus profiler (*KLA Tencor D-600*). Furthermore, the IAI multilayer coatings thicknesses and compositions were characterized by Rutherford Backscattering Spectrometry (RBS) and Particle Induced X-ray Emission (PIXE), with experimental set-ups and analytical possibilities extensively described in the literature^42^. The RBS measurements were performed with a 1.6 MeV ^4^He^+^ ion beam and a Si-diode detector placed at an angle of 0° to the incoming beam direction. The detector measures the energies of the backscattered He ions with an energy resolution of 20 keV. The energies of the backscattered particles depend on the sample’s scattered atoms and depths at which the scattering events occurred. Because the backscattering cross section function is known for each element, a quantitative depth profile can be obtained and fully analyzed with the help of specialized code, e.g. RUMP. PIXE elemental analysis enabled checking the films for unwanted contamination (down to ppm levels). In this experiment a 1.6 MeV ^4^He^+^ beam was used to excite characteristic X-rays of the elements present. The X-rays were detected with a Si (Li) detector of 145 eV resolution placed at 135° to the incoming beam direction. PIXE spectra evaluation was done with the GUPIX code^43^.

The structural properties of the isolated thin film layers and the complete structure of the device were ascertained by XRD using a PANalytical X’Pert PRO equipped with an X’Celerator detector using CuK_α_ radiation at 45 kV and 40 mA, in a Bragg–Brentano configuration. XRD diffractograms were collected over the angular 2θ range 20°–80°, with a scanning step of 0.05°.

Optical properties- reflectance, transmittance and absorption spectra of the samples (glass/thin film or Kapton HN/thin film) were recorded with *JASCO V-770* spectrophotometer for the wavelength range of 200–2500 nm using an integrating sphere. Surface morphology of the coatings were analysed by Scanning Electron Microscope (SEM), in samples coated with gold–palladium alloy in a *Hitachi S2400* apparatus and Atomic Force Microscope (AFM) using a *Witec Alpha 300* RAS confocal spectrometer, with cantilever operated in AC mode with an Al coated probe with a resonant frequency of 75 kHz and spring constant of 2.8 N/m. The lateral and depth resolutions were about 1 nm and 0.3 nm, respectively. Scanning Kelvin Probe Force Microscopy (KPFM) measurements were acquired on an *Asylum Research MFP-3D* standalone AFM system (*Oxford Instruments, Oxford, UK*) operated in room conditions. Measurements were performed in a double pass mode, in which the first pass tracked topography and the second pass tracked the contact potential difference between the AFM probe and the sample while a 3 V AC bias was applied to the probe. Commercially available silicon AFM probes with polycrystalline platinum coated tips (*Olympus AC240TM by Olympus Corporation, Japan, k* = *2 N/m*, *f*_*0*_ = *70 kHz*) were used. Electrical measurements (resistivity-r, type of carriers and their concentration-N and mobility-m) were performed under atmospheric conditions using the *Ecopia HMS-7000* Hall-effect system and the Van der Pauw configuration. The thermoelectric measurements were performed in a homemade apparatus^15^, with and without infrared light. The Seebeck coefficient *S* = ∆*V*/∆*T* values were obtained by measuring the thermovoltage, ∆*V*, as a function of the sample temperature gradient, ∆*T*, and Power Factor (PF) calculated from PF = S^2^/r. The temperature gradient was monitored and measured with an infrared (IR) camera or thermocouple, and the thermovoltage was measured using a nanovoltmeter (*Agilent 34420A*). The samples have a pair of aluminum (Al) contacts (3 mm × 4 mm, 3 mm apart) deposited on the top film of the AZO/IAI structure and on top of the AZO film (as sketched in Fig. 1, the IAI structure covers part of the AZO sample). A thermal gradient was established between electrodes using two independent Peltier modules connected to the respective power sources, one warmed up and the other cooled. The thermoelectric effect was measured on the top of AZO film, at the top of AZO/IAI film and between AZO and AZO/IAI regions in two heating conditions: (1) while uniformly heating the sample and (2) while heating the AZO/IAI region only. For photothermoelectric measurements, the procedure was repeated while the samples were irradiated with infrared, ultraviolet or sun light, with different light intensities (1078 W/m^2^, 540 W/m^2^ and 190 W/m^2^ for UV light and 1240 W/m^2^, 800 W/m^2^ and 380 W/m^2^ for IR light). According to the emission spectrum of the lamps used, the wavelength to which the emission peak corresponds is 1000 nm^44^ for IR and 365 nm for UV lamps^45^. The light irradiated the whole sample or only a part (AZO or AZO/IAI). For this, a cork sheet was placed on the top of the sample, covering a part of interest. For all tests performed, the light was switched ON/OFF and the photovoltage was continuously measured on an *Agilent 34420A* nanovoltmeter connected to a computer, monitoring and recording data. The temperature of the samples was also measured during these tests and kept constant to avoid influence of temperature gradients between contacts. The efficiency of IAI multilayer as *low-e* coatings was demonstrated by comparing the temperature inside a closed cubic box made of normal glass and another similar cube where the normal glass was replaced by a glass coated with IAI layers. Platinum Resistance Temperature Detectors (Pt1000) connected to a computer controlled DataLogger (*Keysight 34972A*) enabled monitoring and recording the temperature inside the boxes for 75 min.

The influence of sample configuration on the thermoeletric voltage was measured with the positive probe (+) always in contact with the electrode of AZO film and the negative probe (−) with the top layer of the AZO/IAI coating. The glass/AZO/IAI structure was uniformly heated, under room temperature (RT) and atmospheric conditions; IAI or AZO sides were alternately heated—the Peltier was placed under the glass/AZO/IAI side to provide heat and the AZO part (without the IAI structure) was left at RT; the temperature difference was applied in reverse; and half of a glass/AZO/IAI sample was heated and the other one cooled.

The photothermovoltage (PTV) was monitored over time and under sun irradiation on samples mounted on a south oriented single-glass window. To verify the influence of different illumination conditions on the PTV, a white paper, black paper and orange Kapton were placed, alternately, between the glass of window and the glass of samples.

## Results

The transmittance and reflectance bandwidth of glass substrates coated with oxide-metal-oxide or dielectric/metal/dielectric (D/M/D) multilayers is controlled through the difference in the refractive index between the dielectric layers and the thin metal layer. The refractive index of dielectric layers is around 2.0 at 580 nm and transmittance is above 90% in visible wavelength range. The optical behaviour of the multilayered D/M/D coatings can generally be adjusted through the metal thickness. A Wolfram Mathematica software package implementing a theoretical simulation model based on the Fresnel equations was used to calculate the transmittance and reflectance spectra of glass/Ag layers of different thicknesses in a previous work^46^. This shows that to improve the transmittance in the visible Ag thickness should be in the range 10–30 nm.

### ITO/Ag/ITO infrared filter

**Figure 2:**
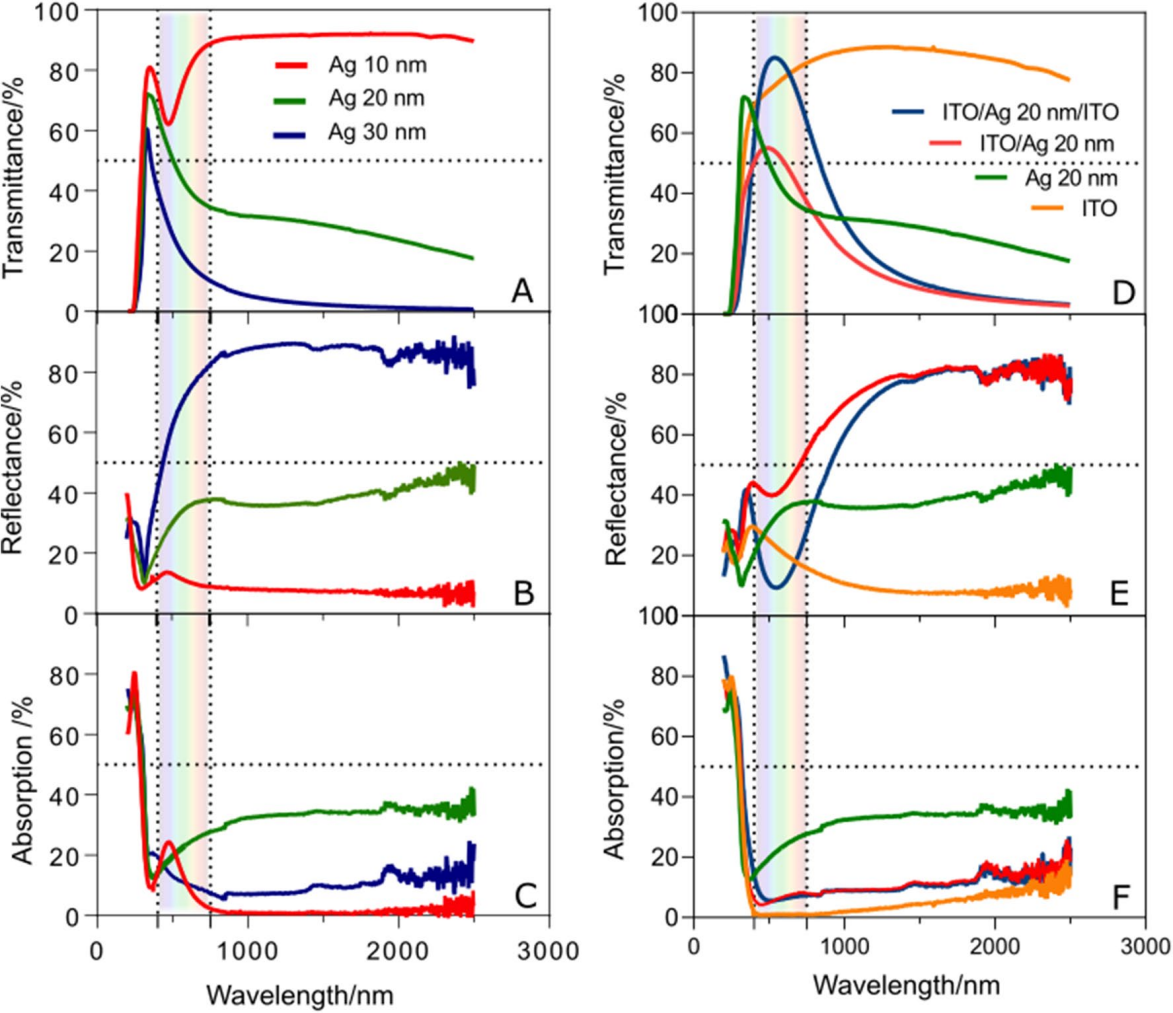
Transmittance, reflectance and absorption spectra of glass/Ag with different thickness of Ag (**A**, **B**, **C**) and glass/ITO, glass/ITO/Ag and glass/ITO/Ag/ITO (**D**, **E**, **F**) samples.

Figure 2 shows the optical spectra of Ag single layers and ITO/Ag/ITO (IAI) multilayers for different Ag thicknesses (10, 20 and 30 nm). As the Ag thickness increases, the near infrared reflectance is enhanced, the band width and transmittance in visible decrease while absorption remains below 40%. The 10 nm thick Ag film exhibits a plasmon absorption peak at ~ 470 nm. This may point to the formation of Ag nanoclusters surrounded by voids as reported previously^47^ which supports surface plasmons. However, this effect vanishes as the film thickness increases, or is placed between two ITO layers, due to coalescence of the nanoclusters and consequent uniformity of films. The optimum Ag thickness that maximizes the IAI structure transmittance in the visible range and keep reflectance above 50% for λ > 1000 nm is 20 nm (Fig. 2 and Figure S1 of supplementary information). This result agrees with simulations^46^ and are consequence of multi reflections at the interfaces and differences in extinction coefficients^48^. The ITO top layer contributes to prevent Ag layer oxidation and minimize the reflection in the visible (as required by low-emission coatings for glass windows applications)^18^. The optical properties are ruled by films thickness, density and stoichiometry, which can be affected by parameters and methods of deposition. Hence, films composition and thickness were assessed by PIXE and RBS (Figures S2 and S3). The composition is consistent with the nominal compositions indicated by the supplier for ITO target and no contaminants were detected in the films: the PIXE spectra (Figure S2) show only the elements In, Sn from the ITO and Ag from the metal layers, along with elements from the substrate (e.g. Si and Ca), no other elements were detected. The analysis of the RBS spectra (Figure S3) was carried out with the known ITO and glass compositions. RBS considers the density of the materials to obtain the thickness, so the difference in thickness obtained by RBS and profilometer for ITO is ~ 30 nm versus ~ 40 nm, likewise for Ag layer (10 nm vs 20 nm), which may indicate the presence of holes, voids or islands in the ITO and Ag films (see supplementary information for RBS measurements). Despite the evidence found for irregularities in the layers these do not seem to significantly impact the optical properties measured. This can be an advantage for applications with e.g. flexible substrates and substrates where irregularities may develop to some extent.

**Figure 3:**
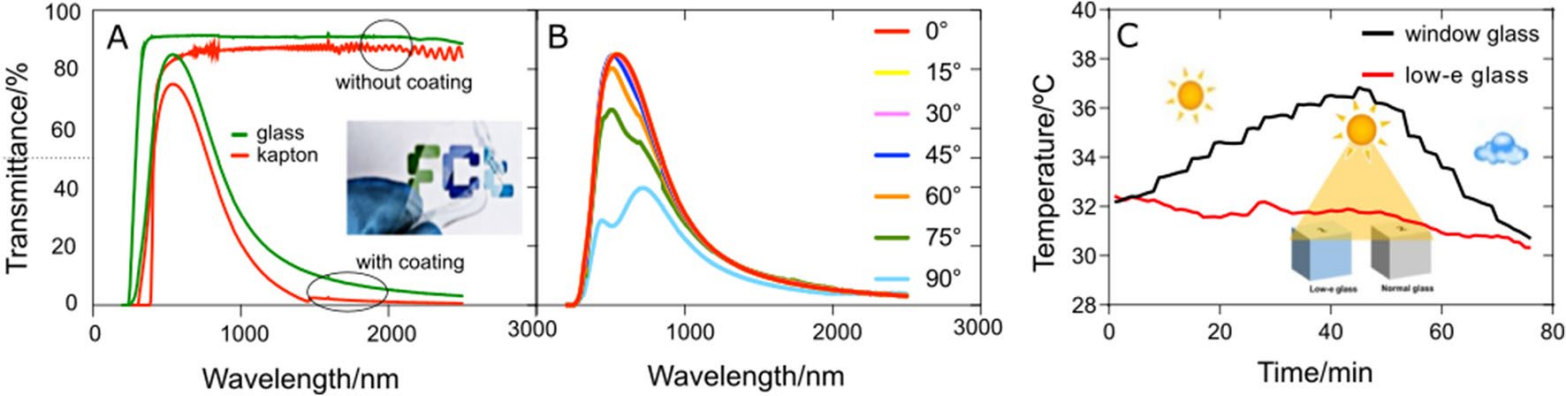
Transmittance spectra of glass or Kapton substrate without and with IAI coating (**A**); glass/IAI for different incidence angles, 0°–90° (**B**) and proof-of-concept—temperature variation within two cubes (ordinary window glass or window glass IAI coated) over time (**C**).

Figure 3A shows that transmittance and reflectance spectra of IAI coatings on polymeric and glass substrates are similar. As such, a *low-e* flexible polymer coating can be an attractive alternative for controlling heat through existing glass windows thus enabling energy savings by reducing air conditioning needs. Considering applications in buildings, low emissivity coated polymeric substrates have advantages compared to glass, as they can be applied on the existing windows without need of replacing glass. Furthermore, the transmittance of IAI coatings is only sensitive at incidence angles above 70°, as shown in Fig. 3B^49^, which is due to film roughness (Figure S4) and a good relationship between thickness and refractive index, since the permittivity of metal layers changes strongly with the thickness^50^.

IAI as a *low-e* coating was tested by comparing the temperature inside two closed boxes in sunlight for a period of 75 min. One box was made of ordinary window glass (WG) and the other of IAI-coated glass (WG-IAI), Fig. 3C. After 40 min of solar radiation, the temperature inside the WG box increased by 4 °C but remained almost unchanged inside the WG-IAI box. When the weather became overcast, the temperature inside the GW-IAI box decreased by 2 °C (from 32 to 30 °C), but dropped rapidly by 6 °C inside the WG box. The observed variation is comparable to that reported by other authors, for example, when using a PDMS coated Al mirror^51^.

### Photothermoelectric (PTE) device

**Figure 4:**
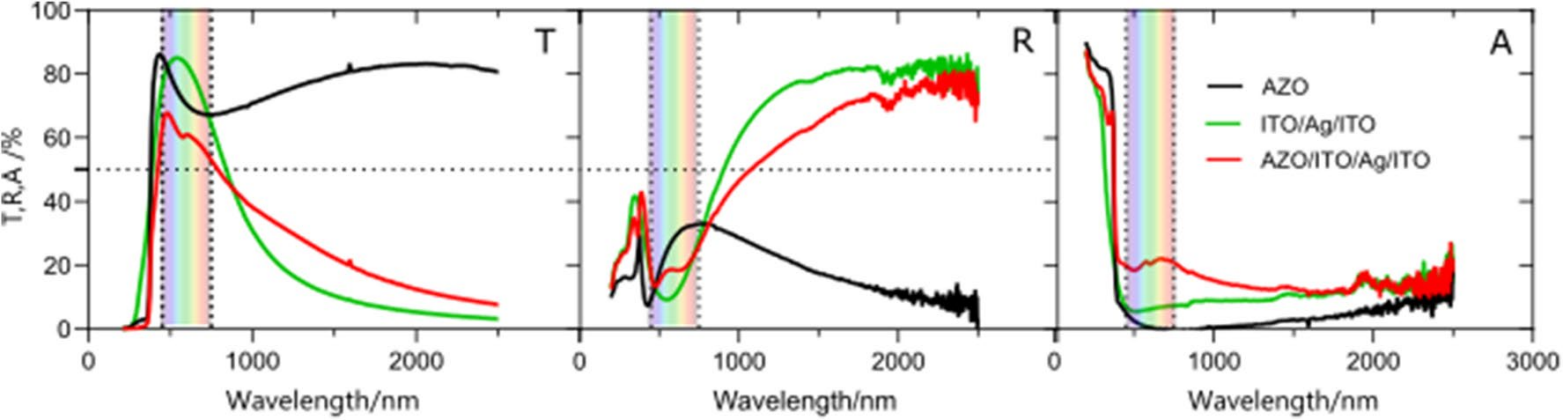
Transmittance (T), reflectance (R) and absorption (A) spectra of AZO, IAI and AZO/IAI layers.

The transmittance (T), reflectance (R) and absorption (A) spectra of the IAI structure (Ag ≈ 20 nm thick) coating the AZO films (≈ 90 nm thick) are shown in Fig. 4. Figure S5 shows the influence of Ag thickness (10–30 nm) on AZO/IAI optical properties. The 30 nm Ag layer lowers the transmittance to ~ 20%, 10 nm improves near infrared absorption and 20 nm improves reflectance. For consistency with previous results, the 20 nm Ag layer was chosen for glass/AZO-IAI samples. Their IR transmittance and reflectance values decreased compared to glass/IAI structure, but absorbance in both visible and IR range increased.

The XRD diffractograms of monolayers and multi-layers show that the single layer of ITO on glass substrate is amorphous, the Ag layer is crystalline and the IAI coating diffractogram exhibits the peaks of crystalline ITO and Ag. However, when it is deposited on AZO, the XRD diffractogram of the thicker AZO layer prevails and the peaks of ITO and Ag vanish (Figure S6).

ITO and AZO are wide band gap materials with high electrical conductivity. The optical gap of ITO (3.78 eV), AZO (4.73 eV), ITO/Ag/ITO (4.82 eV) and AZO/IAI (4.05 eV) was determined by the Tauc’s plot (Figure S7). The electrical and thermoelectric properties measured in planar configuration, as mentioned elsewhere^52^, are shown in Table 1. The Seebeck coefficient and the Power factor of AZO are significantly higher than ITO. Therefore, the thermoelectric effect in planar configuration in IAI or AZO/IAI structures is limited by the thermoelectric performance of ITO layer. ITO has a good electrical conductivity that increases by about one order of magnitude in the IAI structure. In contrast, the AZO/IAI structure exhibits a very high mobility but, lower conductivity than ITO, which means that it is limited by the AZO layer.

**Table 1:** Electrical and thermoelectric properties of ITO, IAI, AZO/IAI and AZO films on glass substrate.

	Electrical conductivity (S/cm)	Hall mobility (cm^2^/Vs)	Bulk concentration (cm^−3^)	Seebeck coefficient (µV/K)	Power factor (µW/mK^2^)
ITO	6.4 × 10^5^	23.3	1.4 × 10^21^	− 7.8	6.4
AZO	3.2 × 10^2^	8.2	3.5 × 10^21^	− 161.7	181.6
ITO/Ag/ITO	5.8 × 10^6^	14.7	2.5 × 10^22^	− 3.8	0.6
AZO/ITO/Ag/ITO	9.5 × 10^3^	48.4	2.2 × 10^21^	− 3.5	1.9

**Figure 5:**
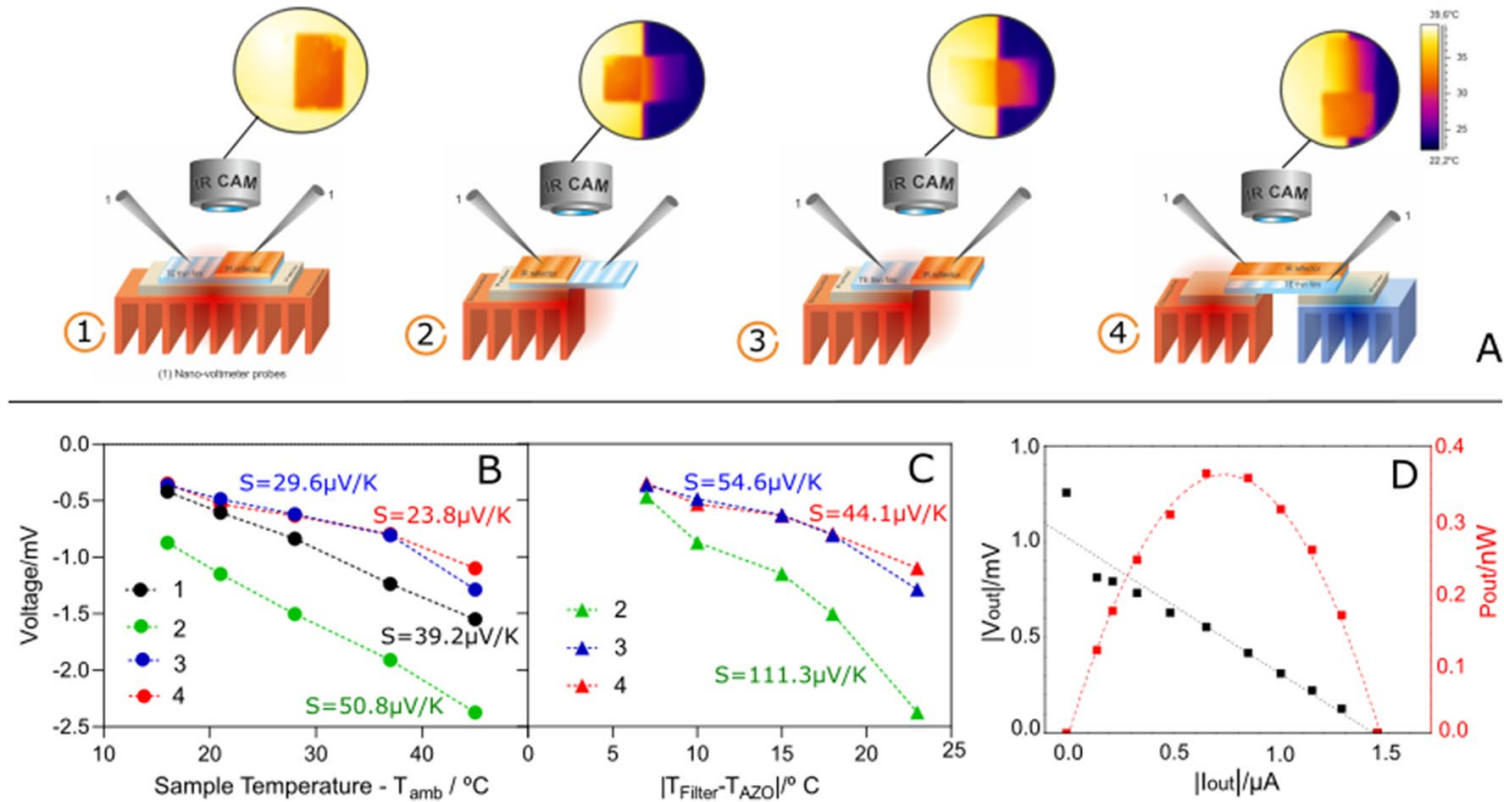
(**A**)Schematics of thermovoltage measurements performed in 4 different configurations and thermal images: (Blue–AZO thermoelectric thin film; Orange—IAI; Red and blue (heating/cooling) heat sinks and Beige—Peltier module): (1) whole sample heated uniformly; (2) AZO/IAI sample heated and AZO on air; (3) AZO heated and AZO/IAI on air; (4) half of the entire sample heated and half cooled; (**B**) and (**C**) Voltage versus difference of temperature between each sample hot and cold side (RT); (**D**) current and Power versus voltage curves of configuration 1.

Due to the high transparency of AZO and reflectance of AZO/IAI in the IR region, it was studied whether under IR radiation there is a voltage between the AZO and the top layer of the glass/AZO/IAI structure (sketched in Fig. 1). For the whole sample irradiated with a red lamp no temperature difference between the AZO and the ITO top layer was evidenced but a voltage around 3 mV was measured. On the other hand while heating the whole sample a Seebeck coefficient of − 31.2 µV/K and power factor of 40 µW/mK^2^ was also measured. The thermoelectric effect was further investigated in different position of contacts as sketched in Fig. 5A. The sample was whole heated (1), only the IAI (2) or AZO (3) regions were heated or half of the sample (4). Despite recording the thermal images from IR camera for each configuration, due to distinct emissivity regions of samples (i.e. between the AZO and IAI top layer) temperature was also measured with a pair of thermocouples. The voltage as function of the temperature difference established between the hot side, warmed up by a Peltier module and the colder side (RT) is shown in Fig. 5B and C. The uniformly heated sample (1) shows an unexpected voltage that increases as the temperature rises. The Seebeck coefficient for configurations 3 and 4 is similar and no temperature difference is observed at the top of either AZO and AZO/IAI sides, hence the potential obtained is due to the IAI structure. A thermal emission of electrons from metal to ITO may occur and, by diffusion due to charge gradient between AZO/IAI and AZO regions, the electrons reach the AZO contact probe. This is corroborated by the results obtained when only AZO/IAI side is heated (2), a huge increase in Seebeck coefficient corresponds to the cumulative effect of configuration 1 and the Seebeck coefficient of the AZO films. This was confirmed by plotting the voltages sum in configuration 1 and 4 or configuration 1 and 3 (Figure S8). In configuration 1, temperature difference measured with a thermocouple on both sides of the sample is similar, reason why Fig. 5C does not show results of this configuration. A representative current–voltage (IV) and power–voltage (PV) curves for AZO/IAI sample heated uniformly are shown in Fig. 5D.

**Figure 6:**
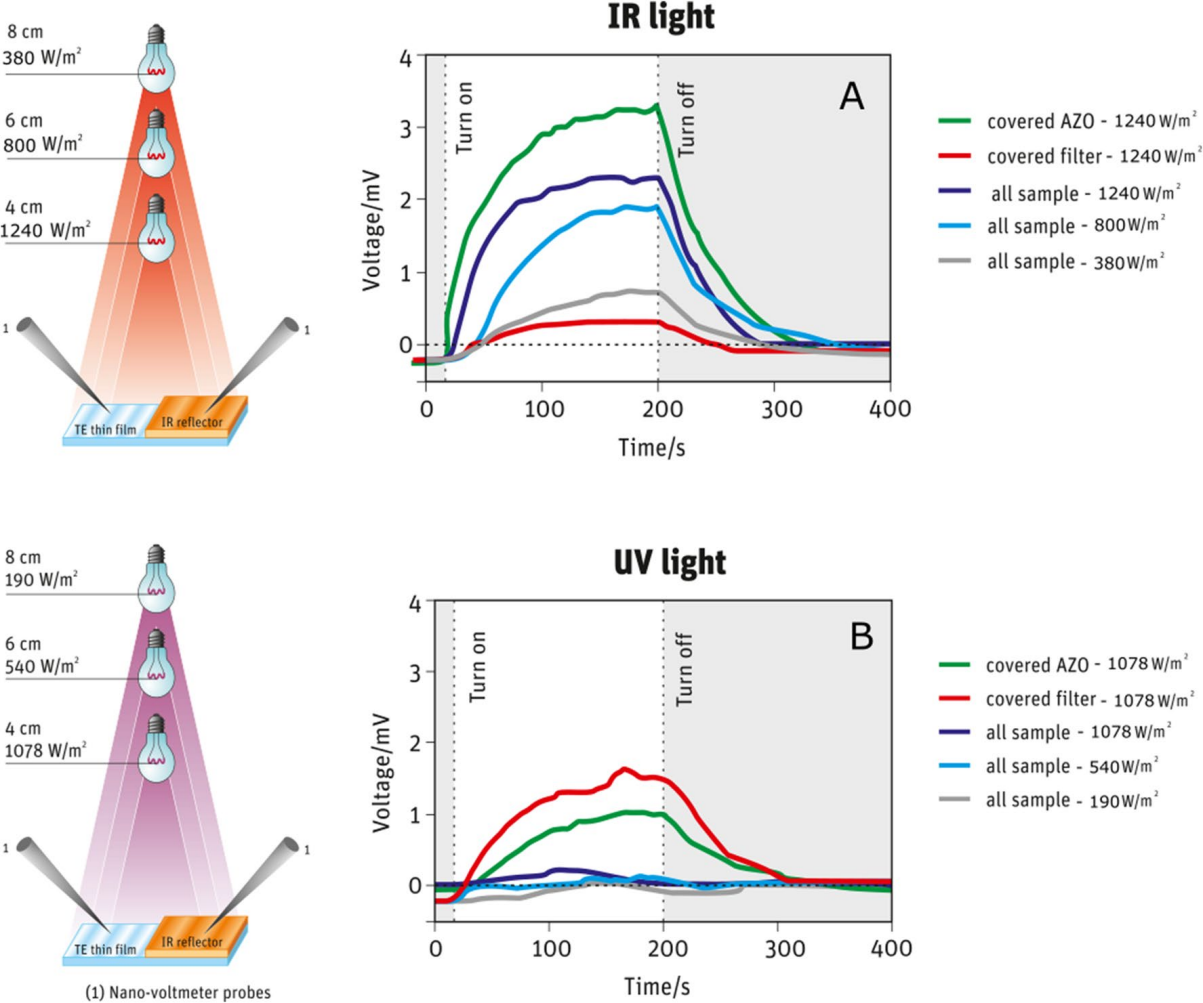
Voltage over time curve for infrared (IR) light (**A**) and ultraviolet (UV)) light (**B**) with different irradiances for UV light (1078 W/m^2^, 540 W/m^2^ and 190 W/m^2^) and for IR light (1240 W/m^2^, 800 W/m^2^ and 380 W/m^2^) while totally or partially irradiating the phothermoeletric device. The lighting scheme was entirely drawn in *CorelDraw* software.

The photothermoelectric effect was observed in the configuration 1, the voltage signal was monitored over time while uniformly irradiating the samples with an IR lamp for three irradiances (1240 W/m^2^, 800 W/m^2^ and 380 W/m^2^), Fig. 6A. Additionally the sample was partially covered with cork to shadow half of the sample (AZO or IAI side). Under IR-light the voltage is proportional to light intensity, which unambiguously reflects a photothermoelectric effect. An empirical linear relationship of V_PTE_ = 1.5 × 10^−2^ P + 1.0 with V_PTE_ in mV and P in mW/cm^2^ fits the obtained results. Radiation incidence in just AZO or AZO/IAI region reveals the contribution of each sample side The results show that the voltage signal is enhanced when the AZO side is covered from light, while it almost vanishes when the glass/AZO/IAI side is covered. As shown in Fig. 3, the absorption of AZO/IAI is about 20% in the visible and 15% in the IR wavelengths (≈ 15% at 1000 nm, the red-light peak^44^) and almost zero for AZO. The percentage of transmittance, reflectance and absorption for wavelengths relating to the emission peak of the used lamps can be found in Table S1. The AZO/IAI IR absorption promotes charges generation that gives rise to a potential difference between this and the AZO region. When the AZO/IAI side is covered keeping the AZO side uncovered, the charge generation disappears and so does the voltage. In contrast, when the AZO side is covered and the AZO/IAI uncovered, i.e. exposed to light, in addition to charge generation, a thermal gradient may occur in the AZO layer due to thermal radiation, which causes an extra thermovoltage and consequently maximizes the signal obtained.

The UV photothermoelectric effect has already been demonstrated for these AZO ALD films deposited on kapton^17^, and is shown in Fig. 6B for films deposited on glass. Under UV-light, there is no voltage signal when the sample is fully irradiated, since charges are generated on both sides of the AZO, the voltage difference is almost zero. UV irradiation of only the AZO/IAI side, or the AZO side, origins a voltage between the irradiated and the non-irradiated part. Higher potential is observed when the AZO/IAI part is covered, caused by the absorption of UV photons and consequent carriers generation. White light is not absorved then no voltage signal was obtained, as expected.

The voltage sign obtained when the sample is heated or radiated with the IR lamp is opposite, while keeping the probes (−) in AZO/IAI and (+) in AZO (Fig. 6). The positive potential means that electrons accumulate on AZO/IAI side. Therefore, absorption of photons generating free charges is consistent with the voltage obtained.

**Figure 7:**
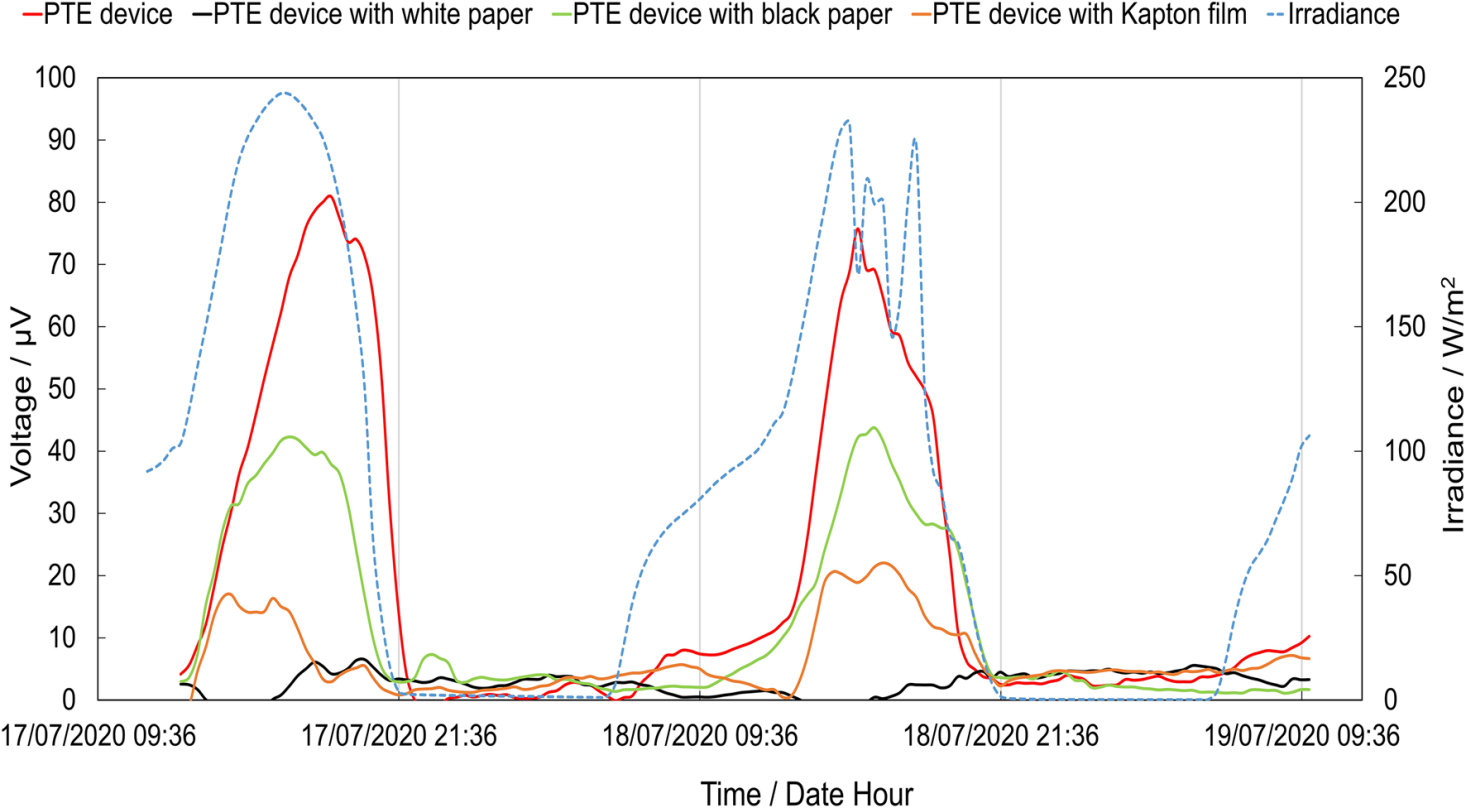
Voltage and irradiance variation over time of photothermoelectric device glued to the window. Blue line—irradiance, red line—AZO/IAI sample, green line—AZO/IAI completely covered with a black paper, orange line—AZO/IAI covered with Kapton film and black line—AZO/IAI covered with white paper.

The samples were mounted on a glass window, south oriented, for continuously monitoring their photothermoelectric performance under sun radiation. The measured voltage over time shows a clear dependence with the sun-light intensity (Fig. 7). The maximum voltage appears for the irradiance peaks at 12:00 PM–15:00 PM and overnight it drops to a voltage near zero. The multi-layer device photothermoelectric response trend to the solar irradiance is similar to that obtained with the light source bulbs (UV and IR), unlike the voltage amplitude is lowered, from millivolts to microvolts due to lower irradiance.

As the solar spectrum includes UV, VIS and IR radiation (and heat radiation), the influence of each component to the observed voltage was further investigated. Hence, in this scope, white paper, black paper or kapton film were used to act as filters for the incoming solar radiation. These were alternatively placed between the glass window and the sample, with the AZO/IAI top layer facing the selected ‘filter’. The white paper reflectance spectrum, and the black paper and Kapton film absorption spectra are shown in Figure S9. While the white paper shows a very high reflectance (i.e. > ~ 80%) over the entire wavelength range (preventing sample illumination or heating), the black paper shows a very high absorbance (i.e. > ~ 80%). The kapton film absorbs in a narrower wavelength range, in the UV range. Throughout the use of each filter, the sample voltage over time was monitored during 2 days as shown in Fig. 6. For reference, this also includes the sample voltage–time curve for a glass/AZO/IAI sample directly exposed to sun light (without the use of any filter). As expected, the voltage signal is near zero when reflecting the sun light with white paper. When using the Kapton film as filter it decreases from 80 to 20 µV due only NIR absorption. This proves that the UV component has an important contribution to the voltage signal generated under solar radiation. The optical interactions with the sample are removed with black paper but have thermal interacions because the device heats up more on the paper side and we have the influence of a thermail gradient in the sample. Therefore, the generated voltage is related to the thermoelectric effect described in Fig. 5 configuration 1. The results clearly show that AZO/IAI coating can work as a thermoelectric and photothermoelectric device. To this phenomenon a possible photothermal effect caused by a metal semiconductor junction is possible, as the Kelvin probe force microscopy (KPFM) reveals that the potencial difference between AZO and AZO/IAI sides is about 0.2 V, (Figure S10). However the potential measured experimentally between both regions is almost zero, eliminating this hypothesis. Thus, injection of electrons from metal into ITO layer and generation of electron–hole pairs are the most probable mechanisms behind this effect. Hot-ejection charge from metal layer can occur by several process as described by Khurgin^53^, and surface collision assisted decay is expected to occur in a very thin layer of the metal thickness, giving electrons to oxide layer. Other works report the PTE effect based on surface plasmon of nanoparticles or nanowires, which is also possible in a very thin metal layer sandwiched between oxides layers^36,54–57^.

Although, PTE effect has been correlated with the generation of thermal gradient caused by punctual heating, plasmonic effects were recently demonstrated using nanostructures to enhance light absorption in metallic layers^58^ or a ballistic thermal injection in Au/CdO structures^54^.

Indeed, recently Dai et al.^58^ have demonstrated infrared photothermoelectric voltage on the LaAlO_3_/SrTiO_3_ interfaces when irradiating the Au/Ti metal contact with 1.5 eV energy, which is below the oxide bandgaps, correlating that with photoexcitation of hot carriers in the metasurface contacts and the thermoelectric charge separation by interfacial two-dimensional electron gas (2DEG). A sensitivity of 4.4 V/W at room temperature was reported. They observed a positive photovoltage signal near Au/Ti anode electrode and negative near the cathode, meaning that electrons flow from the metal to the oxide. As the photoexcited electrons relax to ground state the emission of phonons and the electrons scattering produces a local heating which originates a local thermal gradient. The photothermoelectric effect on bulk SrTiO_3_ (STO) material with a responsivity up to 1.2 V/W at 10.67 µm photon radiation of Ag surface was reported by Lu et al.^59^. The voltage signal was measured between two Ag electrodes separated by about 10 mm length, 0.5 mm width and 0.11 mm thickness. The maximum signal occurs at STO/metal interface when laser spot shines the respective Ag electrode, therefore also related to the photo excitation of Ag electrons. Plasmon-induced and photo-excitation carriers have been distinguished by Zheng et al.^60^ by comparing the Au/TiO_2_ Schottky contact and Au/Ti/TiO_2_ ohmic contact. They concluded that for Schottky barrier with a barrier height of around 1.1 eV, or ohmic contacts, the nonresonant photocurrent in plasmonic nanowires is due to interband transitions. Moreover, in ohmic devices photocurrent results from direct excitation and not plasmon decay, as photocurrent is independent of the geometry and polarization of incident light. Zheng et al. observed a responsivity of about 60 nA/mW. A ballistic thermal injection mechanism in Au/CdO junctions was reported by Tomko et al.^54^. Their experiments were conducted to demonstrate that in Au/CdO junctions the excited electron of Au stay at highly elevated temperatures for prolonged time due to the weak electro-phonon coupling factor of Au, and that the energy of excited electrons can ballistically traverse the Au film reaching the Au/CdO interface and directly couple the excess of energy into CdO electrons, without charge transfer. The authors called it a ballistic thermal injection (BTI).

According to the BTI phenomena, silver atoms of the IAI structure become excited with NIR photoexcitation and the Ag layer overheats, as a result. The overheating of metal can reach 2 K^58^. If so, this will enhance temperature on the IAI/AZO side, promoting electrons to flow towards the positive contact in AZO leading to a negative ΔV. But this electrons flow is also occurrs when hot carriers are injected into ITO and AZO sides. Therefore, from the obtained results it is not possible to distinguish which mechanism prevails when the PTE device is uniformly heated. Heating only the IAI side of sample it seems to have the previous effect coupled to a pure thermoelectric effect as the ΔV increases. In contrast, heating only the AZO part, reduces the ΔV by the same value that was added in the opposite situation. The photothermoelectric voltage obtained by sample irradiation with solar spectrum shows a responsivity of about 8 mV/W, which is below other results found in literature for nanostructured devices. Nevertheless the combination of low-e coatings with photothermoelectric effect was never tried. Further exploration will certainly broaden possible applications and provide new ways of improving the proposed devices.

## Conclusions

ITO/Ag/ITO (IAI) coatings were optimized to have high reflection for near infrared radiation. Ag layer of 20 nm thickness provides the best results, with 80% of transmittance in the visible range and reflection of 50–80% in the near infrared range. The efficiency of these coatings for energy saving through control of IR radiation in a glass windows was proved by comparing the temperature inside closed glass boxes with and without the ITO/Ag/ITO coating. After 40 min of sun exposure while the temperature inside the both boxes have a difference of 4 °C, higher for uncoated glass box. A combined photo and thermoelectric effects was also demonstrated in AZO films coated with ITO/Ag 20 nm/ITO layers under light and uniform heating. A voltage response is proportional to the solar irradiance as observed on samples placed in a glass window facing south continuously measured for 2 days. Overall, a glass/window coated with thermoelectric transparent thin films such as AZO and low-e coatings as the IAI structure can improve energy efficiency of buildings as well as convert thermal and photonic energy into electrical energy.

## Supplementary Information

Supplementary Information.
