# Supplementary material for: Near infrared photothermoelectric effect in transparent AZO/ITO/Ag/ITO thin films
Source: Sci Rep. 2021 Dec 21;11:24313. doi: 10.1038/s41598-021-03766-y (PMC8692428; doi:10.1038/s41598-021-03766-y)
Supplement: Supplementary file 1 — Supplementary Information. [file 41598_2021_3766_MOESM1_ESM.pdf]

Near infrared photothermoelectric effect in transparent AZO/ITO/Ag/ITO thin films

C. BIANCHI[a] *, A. C. MARQUES[a], R. C. DA SILVA[b], T. CALMEIRO[a] AND I. FERREIRA[a]

[a] *CENIMAT/I3N, Department of Materials Science, NOVA School of Science and Technology, Largo da Torre, 2829-516 Caparica, Portugal*

[b] *IPFN-IST/UL, Instituto de Plasmas e Fusão Nuclear, Instituto Superior Técnico, Universidade de Lisboa, Estrada Nacional 10, 2695-066, Bobadela, Portugal*

[*] *e-mail: cb.marques@campus.fct.unl.pt*

Supplementary information

Optical spectra of ITO/Ag/ITO structure

Figure S1 shows the influence of Ag thickness on the transmittance, reflectance, and absorption of glass/ITO/Ag/ITO. The 20 nm thick Ag thin film shows a better compromise between high transparency in the visible and high reflection in the infrared range.

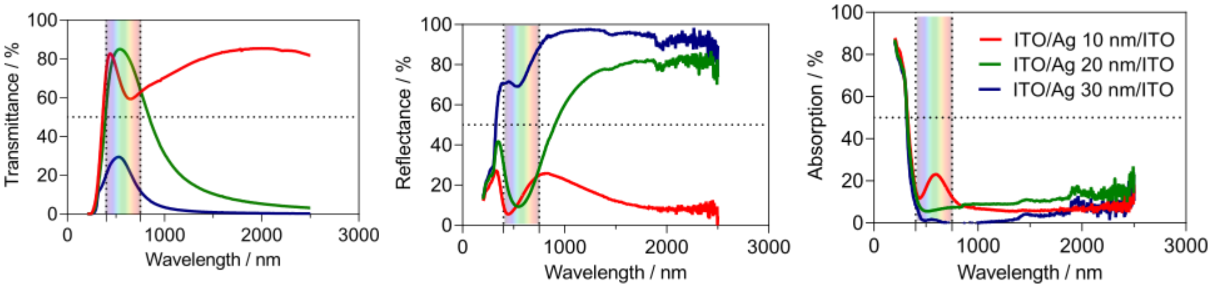

Figure S1 - Transmittance, reflectance, and absorption of IAI coatings with different Ag layer thickness (10, 20 and 30 nm).

Elemental analysis of IAI multilayers

Helium ions are less efficient at exciting X-rays from materials, due to their lower excitation cross-sections and lower penetration in the matter: the excitation of elements in the substrate is much weaker, providing a better separation of the X-rays emitted by the elements in the film. Figure S2 shows that the contributions of In and Sn are clearly visible in the 40 nm thick ITO film and 20 nm IAI structure deposited onto glass, while that of Ag is detected only in the IAI structure. Spectral analysis with the GUPIX software further shows that the corresponding In-Sn concentration ratios are similar in both films. The Ca and Si contributions (the latter being well apart at 1.74 keV, and thus not shown in Figure S2) belong to the glass substrates; no other elements were detected. In addition, the substrate was further investigated through 2 MeV proton beams but only substrate glass elements were detected. Therefore, one can conclude that these samples have no contaminants at detectable levels.

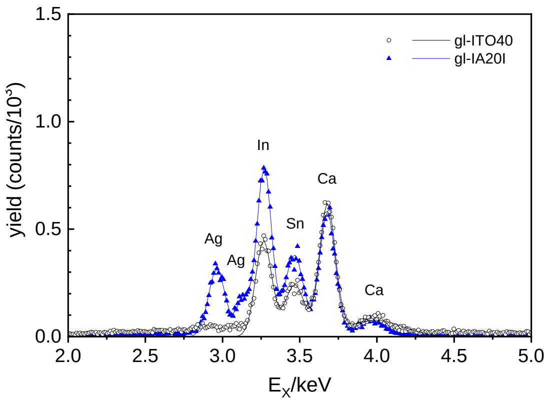

Figure S2 - PIXE spectra from samples of ITO and IAI deposited onto glass (gl-ITO40 and gl-IA20I, respectively), ITO and Ag having nominal thickness of 40 nm and 20 nm respectively.

The compositions found by PIXE are consistent with the nominal compositions indicated by the supplier for ITO target. As such analysis of the RBS spectra were carried out with the compositions known for ITO and the glass used as substrate. Figures S3 depicts the RBS spectra obtained from two samples: 'gl-ITO40' made by deposition of nominally 40 nm thick ITO onto glass, and 'gl-IA20I' made by deposition of nominally 40 nm thick ITO onto glass, followed by a sequential deposition of nominally 20 nm thick Ag and 40 nm thick ITO over layers. Spectra in Figure S3 A were collected under normal incidence of the ion beam, while spectra in Figure S3 B were collected under tilted incidence, at high exiting angle (10° take-off from the surface), to gain sensitivity on the state/quality of the interfaces and check the thicknesses. The full lines represent the simulations that best fit the experimental data as calculated with the RUMP code. For each sample a unique set of compositions-and-thicknesses adequately reproduce all corresponding spectra. Although the compositions found are as specified by the manufacturer for the target material, the thicknesses found deviate from the nominal values. However, an important aspect to bear in mind is that RBS does not measure true thicknesses (i.e. physical thicknesses). Rather, it measures areal densities (atoms/cm^2 or μg/cm^2), which can be converted into physical thicknesses once densities are known (atoms/cm^3 or g/cm^3). Notice that local inhomogeneities and structural irregularities, such as holes, voids, and islands, affect density and thus the physical thickness reported. As such, discrepancies between thicknesses reported from the analysis of RBS results and those obtained by methods of direct measurement of physical thicknesses provide strong evidence for the occurrence of irregularities like inhomogeneities and/or structural defects – holes, voids, islands, etc. – in the analysed materials. The case is that for ITO layers sitting on the glass substrate, the surface densities (atoms per cm^2) calculated from analysis of the RBS spectra correspond to physical thicknesses (~30 ± 2 nm), that are smaller than measured with the stylus profilometer (40 ± 3 nm) if nominal density of 7.14 g/cm^3 (or 7.82×10^{22} atoms/cm^3) is taken for ITO; likewise for Ag atop this layer (~10 ± 1 nm vs. 20 ± 4 nm). It is as if the media densities are smaller than in the bulk materials. This may be an indication of holes, voids, or islands in the deposited films. On the contrary the top ITO layer (in gl-IA20I) shows apparently thicker than nominal (48 ± 4 nm vs. 40 ± 3 nm). This effect may be explained by filling of the underlying voids and cavities: in such case the beam ions effectively probe a larger areal density. Furthermore, part B of Figure S3 clearly shows that the RBS spectra change markedly upon large tilts, more so for the thicker gl-IA20I sample: the spectral contribution from the ITO elements broadens and its low energy flank spreads significantly. Although the fit model still describes adequately the main features of the experimental data – spectral intensities and energy profile widths – overall agreement requires that an energy dependent spread is allowed for to get a better fit. Such spread,

increasing as energy decreases is consistent with rough interfaces, comprising irregularities (holes, voids, islands, etc.) that introduce extra scattering and energy spread of the beam particles.

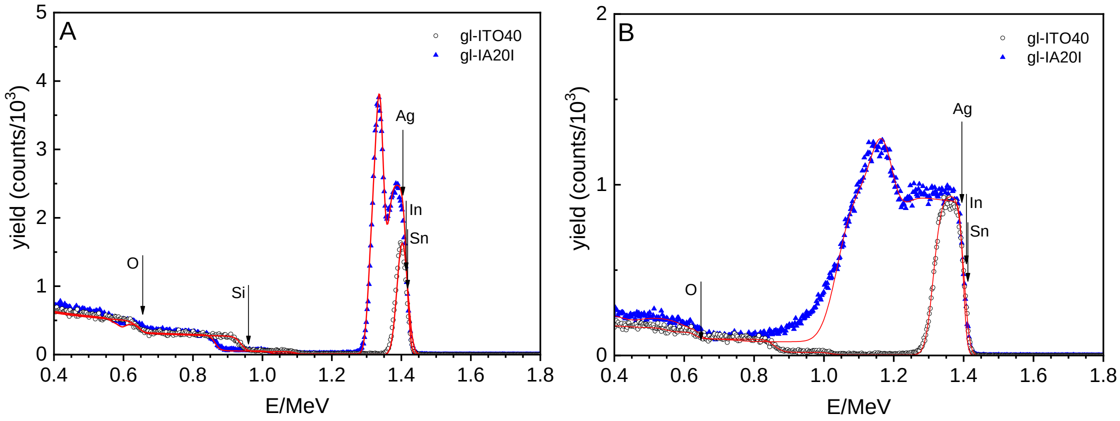

Figure S3: RBS spectra from samples of ITO and IAI deposited on glass (gl-ITO40 and gl-IA20I, respectively) under 0º (A), and 40° (B) incidence (exit angle 80º), ITO and Ag having nominal thicknesses of 40 nm and 20 nm, respectively. Notice (in B) the significant spectral broadening and the particularly large spread of the low energy flank (0.9-1.1 MeV) in the case of the thicker gl-IA20I sample. Simulation/fit curves are ploted with red solid lines and the expected positions of the elements (sitting at the surface) are indicated by arrows.

Morphology of silver layer and IAI multilayer

Additional information about the influence of Ag thickness on the surface morphology of glass/ITO/Ag/ITO top layer and Ag layer can be observed in the SEM images of Figure S4 (first two columns) and compared with the surface roughness of Ag film with the same thickness deposited on glass substrate. The AFM analyses of Ag layers on glass reveal a smooth surface independent of thickness. The root mean square roughness (RMS) values are ~1.4 nm, ~2.1 nm and ~1.8 nm for 10 nm, 20 nm and 30 nm of Ag thickness, respectively. However, the top raw of SEM images show that samples with an Ag layer of 10 nm have several agglomerates with possible empty spaces in between. This was also observed in previous work, the very thin Ag nucleates in small clusters and then the films start growing from there [1, 2]. Indeed, SEM images of 20 nm and 30 nm Ag films show very small grains but form a like-continuous film. This agrees with the transmittance values presented in Figure S4.

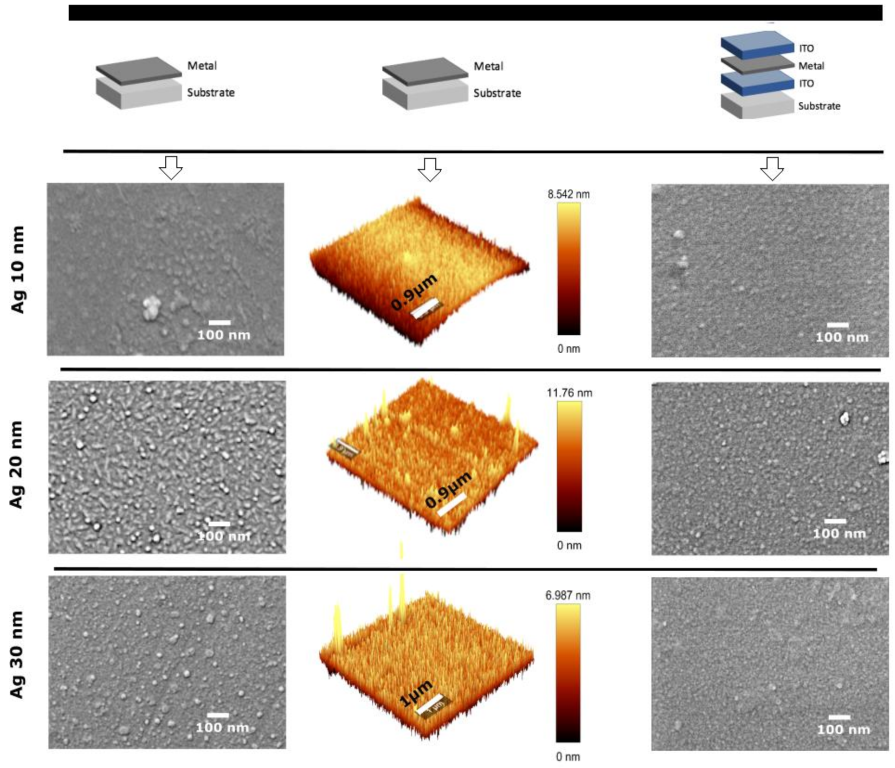

Figure S4 – Influence of Ag thickness in the surface morphology of glass/ITO/Ag/ITO top layer and glass/Ag samples. SEM images display the surface morphology of the top ITO layer in the IAI structure, and the AFM maps show the surface roughness of Ag layers deposited on glass.

Optical spectra of AZO/ITO/Ag/ITO structure

The influence of Ag thickness on the transmittance, reflectance and absorption of glass/AZO/ITO/Ag/ITO samples is shown in Figure S5. The most important evidence is a drastic decrease in transmittance for Ag thickness around 30 nm corresponding to a reflector behaviour of this structure. For the 20 nm Ag thickness, the one used in the thermoelectric studies, the structure has a low reflectance in the visible region of the spectra which increases for infrared region, and the transmittance has the opposite behaviour (high in the visible range and low in the infrared region). The absorption is ~20% in the visible range and ~10% in the infrared range.

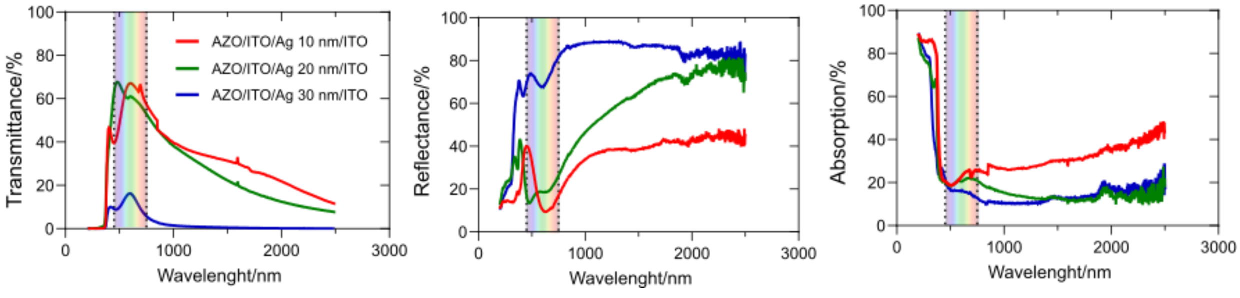

Figure S5 - Transmittance, reflectance and absorption of glass/AZO/IAI with three different thickness of Ag layer (10, 20 and 30 nm).

X-ray Diffraction (XRD)

The XRD diffractograms of monolayer and multi-layered films deposited on glass substrate are shown in Figure S6. The XRD diffractograms show that the ITO thin film is amorphous, which would be expected since ITO for very thin layers deposited on glass without annealing has an amorphous structure [3,4]. The Ag layer shows a main peak located at 2θ = 38.02° corresponds to the (111) planes, characteristic of polycrystalline silver even for nanometer layers or grains [1,5]. According to the Joint Committee on Powder Diffraction Standards (JCPDS) card No. 036-1451, AZO thin film has a hexagonal wurtzite structure with *P63mc* space group with the three major diffraction peaks corresponding to the (100), (002) and (101) planes [6]. Regarding AZO and IAI coated AZO, the diffractogram shows only the AZO peaks and the characteristic silver peak is no longer visible. This is because the silver layer is very thin compared to the two ITO and AZO layers. Although a single layer of ITO is amorphous, the IAI structure shows two main peaks, one clearly identified as the (111) silver peak and a second at 2θ = 35.17º attributed to (400) ITO crystalline film planes [7]. At first glance it could also be attributed to Ag_2O, related to some oxidation of the Ag layer before or during the ITO deposition. However, these generally lie at $2\theta_{Ag_2O}$ = 38º - 38,6º or $2\theta_{AgO}$ = 37° [8,9].

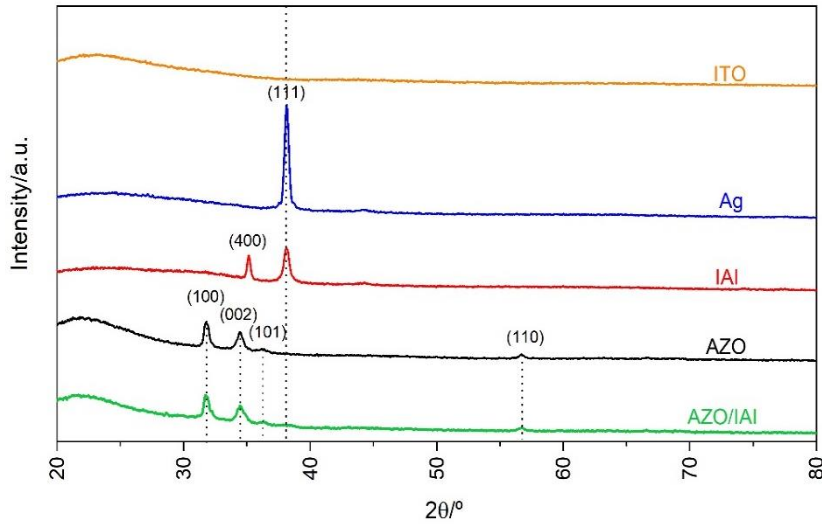

Figure S6 – XRD diffractograms of ITO, Ag, ITO/Ag/ITO and AZO thin film deposited on glass and the final structure (AZO/ITO/Ag/ITO) deposited on glass.

Optical band gap

The band gap was determined for the ITO and AZO individual layers and for the multilayer IAI structures on glass (with and without AZO layer). The graphs of Figure S7 shows the plot of $(\alpha h\nu)^2$ versus photons energy and the respective linear regression considered for obtaining the optical band gap from Tauc's plot [10], $(\alpha h\nu)^{1/n} = A(h\nu - E_g)$, where h is the Planch's constant, ν is the photon's frequency, α is the absorption coefficient, E_g is the optical band gap and A is a proportionality constant. Depending on the optical absorption strength the band gap can vary

with n = 1/2 for direct allowed transitions, n = 3/2 for direct forbidden transitions, n = 2 for indirect allowed transitions, and n = 3 for indirect forbidden transitions. For oxide materials the transitions are mostly direct allowed transitions.

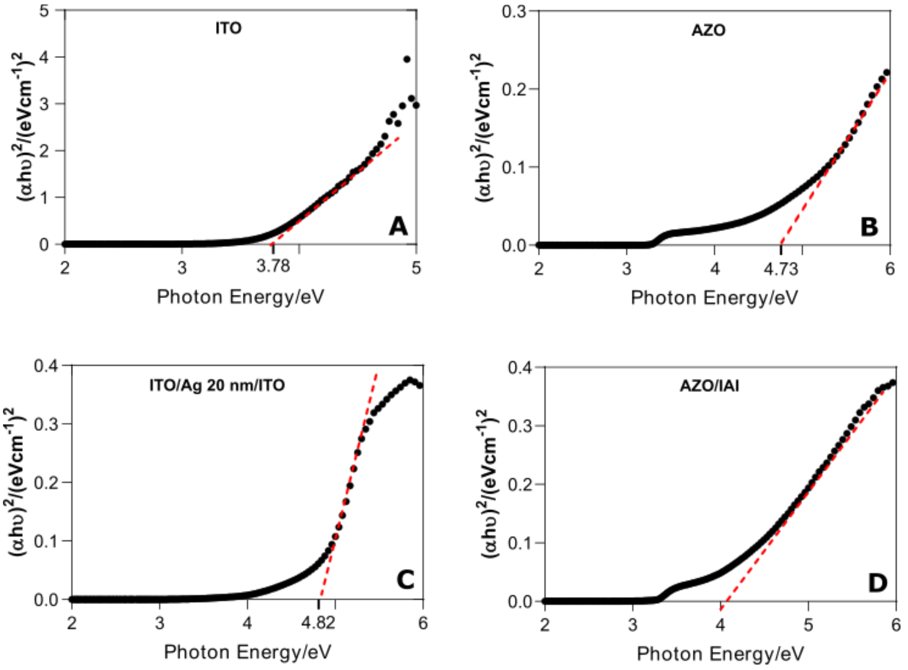

Figure S7 - Tauc's plot of ITO (A), AZO (B), IAI (C) and AZO/IAI (D) samples.

Optical properties of AZO and AZO/ITO/Ag/ITO structure

Figure S8 shows the potential for configuration 2 (shown in figure 6 A) and the sum voltage corresponding to configurations 1 and 3, and likewise for configuration 1 and 4, evidencing that configuration 2 combines the thermoelectric effect (in configuration 3 or 4) with configuration 1.

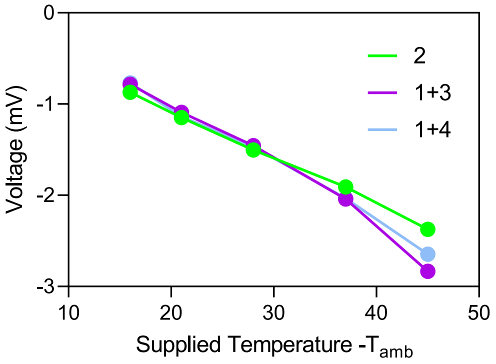

Figure S8– Potential versus temperature of substrate for configuration 2 (AZO/IAI sample heated and AZO at RT); sum of voltage of configuration 1 (whole sample heated uniformly) and 3 (AZO heated and AZO/IAI at RT and 1 + 4 (half of the entire sample heated and half cooled).

Optical properties of AZO and AZO/ITO/Ag/ITO structure

Table S1 - Transmittance, reflectance, and absorption of AZO and AZO/IAI samples, for the wavelengths corresponding to the emission peaks of the IR (1000 nm) and UV (365 nm) lamps used in the light test chamber.

	$\%T_{365\,nm}$	$\%R_{365\,nm}$	$\%A_{365\,nm}$	$\%T_{1000\,nm}$	$\%R_{1000\,nm}$	$\%A_{1000\,nm}$
**AZO**	5	22	73	71	29	0
**AZO/IAI**	3	29	68	38	47	15

Optical properties of the materials used to filter the light: Black/white papers and Kapton film

White and black papers as well as Kapton film were used to reflect, absorb or filter the sun radiation on the samples of Figure 7. The reflectance spectra of white paper, the absorption spectra of black paper and the absorption band of Kapton film were measured and shown in the Figure S9. These confirm a high reflectance of white paper, and a high absorption for the black paper, both in the entire spectral region, and high absorption in the UV region for the Kapton film (for higher wavelengths, *i. e.* after 400 nm, the absorption is almost null).

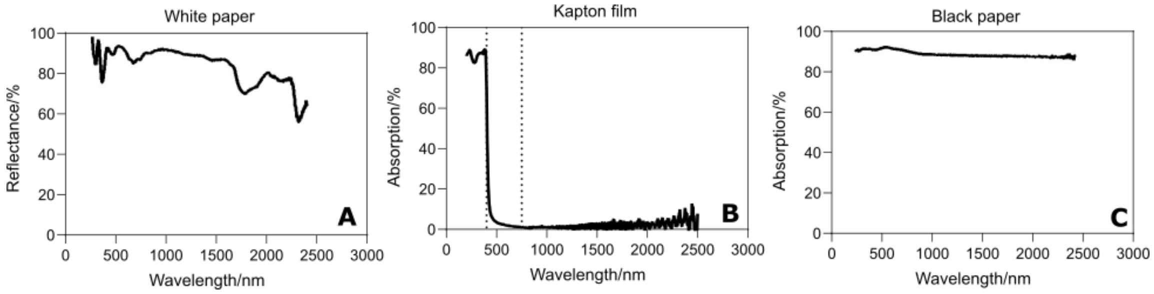

Figure S9 – Reflectance of white paper (A), absorption of Kapton film (B) and black paper (C).

Kelvin probe force microscopy (KPFM)

Kelvin probe force microscopy allows obtaining surface topography, morphology, roughness, surface potential and phase without contacting the sample [11]. The obtained maps for the individual samples and multilayers IAI and AZO/IAI are shown in Figure S10. KPFM measures a contact potential difference (CPD) between the sample surface and the tip, that is, the difference between the work-function of the material and the probe [12]. Therefore, it is possible to check if the surface potential is uniform all over the surface and compare it between samples to understand any possible difference of work-functions. The CPD map of individual AZO, ITO, and Ag layers are quite different, it ranges between 0.28 V and 0.36 V for AZO, 0.09 V and 0.17 V for ITO and -0.17 V and -0.1 V for Ag. For the multilayers ITO-Ag-ITO CPD ranges between -0.172 V and -0.1 V while for AZO/ITO-Ag-ITO is between 0.07V and 0.15 V. Thus, IAI structure CPD seems to be dominated by the Ag work function and AZO-IAI by the ITO work function. Due to work-function differences between AZO and AZO/IAI sides was expected a Schottky like-barrier in the range of 0.21V, also in agreement with expected work-function of silver, around

4.3-4.7 eV [13] and AZO or ITO [14] between 4.4-4.7 eV [14 15]. The maps also confirm the uniformity of films either compositions (given by phase) or surface roughness (given by topography).

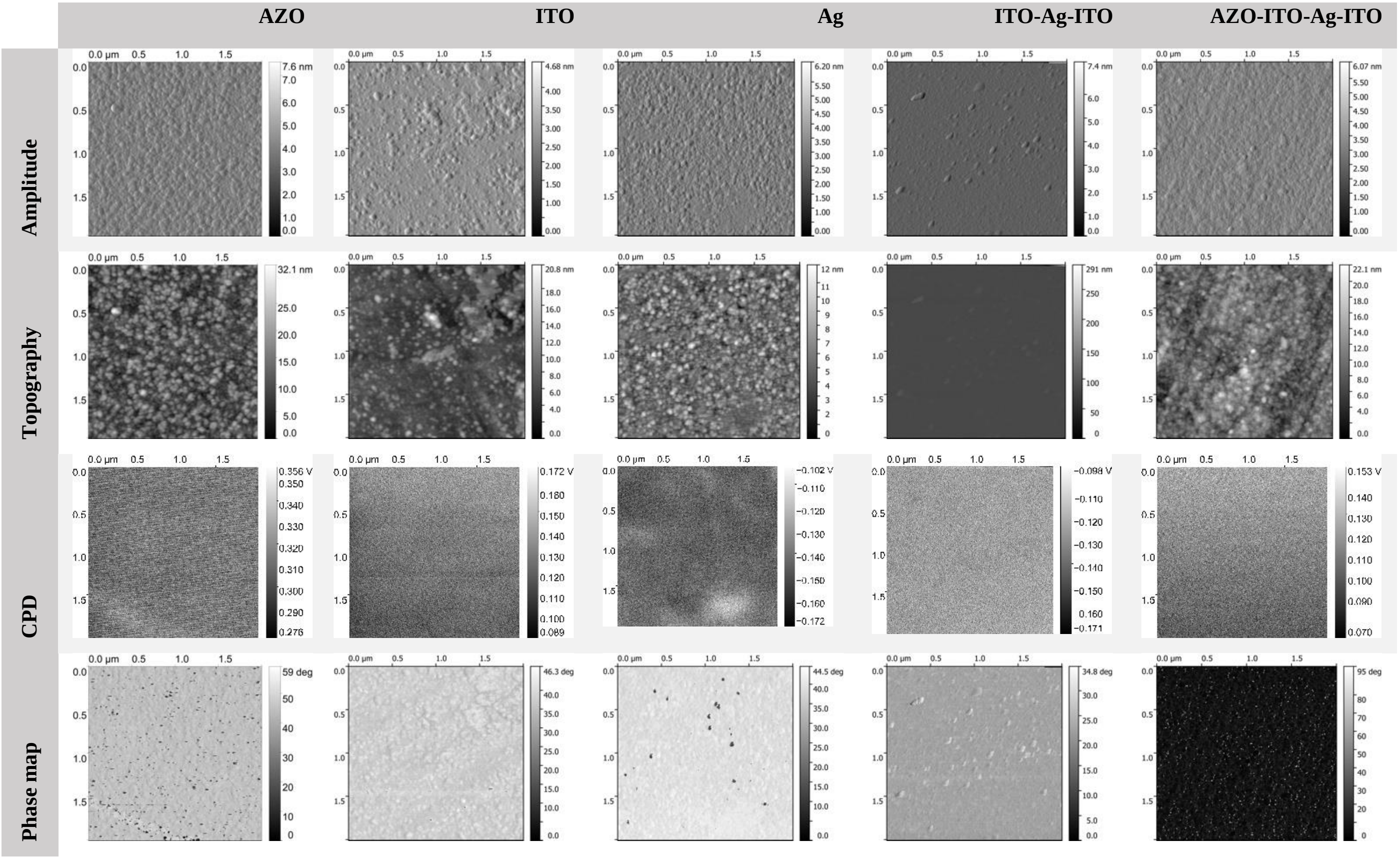

Figure S10 – Kelvin probe force microscopy performed on individual AZO, ITO, Ag layers and IAI, AZO/IAI multilayers, and the corresponding amplitude, topography, contact potential difference (CPD) and phase map.

References

1. Gaspar, D. *et al.* Ag and Sn Nanoparticles to Enhance the Near-Infrared Absorbance of a-Si:H Thin Films. *Plasmonics* **9**, 1015–1023 (2014).
2. Gaspar, D. *et al.* Influence of the layer thickness in plasmonic gold nanoparticles produced by thermal evaporation. *Sci. Rep.* **3**, 3–7 (2013).
3. Fan, Z. *et al.* Hydrogen plasma exposure of In/ITO bilayers as an effective way for dispersing In nanoparticles. *ACS Publ.* (2017).
4. Mazur, M. *et al.* Effect of thickness on optoelectronic properties of ITO thin films. *Circuit World* **ahead-of-print**, (2020).
5. Rohom, A. B., Sartale, S. D., Garg, A. B., Mittal, R. & Mukhopadhyay, R. Deposition and Characterization Of Nanocrystalline Silver Thin Films By Using SILAR Method. in 397–398 (2011).
6. Tsay, C.-Y. & Hsu, W.-T. Comparative Studies on Ultraviolet-Light-Derived Photoresponse Properties of ZnO, AZO, and GZO Transparent Semiconductor Thin Films. *Materials* **10**, 1379 (2017).
7. Shigesato, Y., Koshi-ishi, R., Kawashima, T. & Ohsako, J. Early stages of ITO deposition on glass or polymer substrates. *Vacuum* **59**, 614–621 (2000).

8. Saroja, G., Vasu, V. & Nagarani, N. Optical Studies of Ag2O Thin Film Prepared by Electron Beam Evaporation Method. *Open J. Met.* **03**, 57–63 (2013).
9. Al-Kuhaili, M. F. Characterization of thin films produced by the thermal evaporation of silver oxide. *J. Phys. Appl. Phys.* **40**, 2847–2853 (2007).
10. Viezbicke, B. D., Patel, S., Davis, B. E. & Birnie, D. P. Evaluation of the Tauc method for optical absorption edge determination: ZnO thin films as a model system. *Phys. Status Solidi B* **252**, 1700–1710 (2015).
11. Yasutake, M., Aoki, D. & Fujihira, M. Surface potential measurements using the Kelvin probe force microscope. *Thin Solid Films* **273**, 279–283 (1996).
12. Melitz, W., Shen, J., Kummel, A. C. & Lee, S. Kelvin probe force microscopy and its application. *Surf. Sci. Rep.* **66**, 1–27 (2011).
13. Dweydari, A. W. & Mee, C. H. B. Work function measurements on (100) and (110) surfaces of silver. *Phys. Status Solidi A* **27**, 223–230 (1975).
14. Park, Y., Choong, V., Gao, Y., Hsieh, B. R. & Tang, C. W. Work function of indium tin oxide transparent conductor measured by photoelectron spectroscopy. *Appl. Phys. Lett.* **68**, 2699–2701 (1996).
15. Chen, T. L., Betancur, R., Ghosh, D. S., Martorell, J. & Pruneri, V. Efficient polymer solar cell employing an oxidized Ni capped Al:ZnO anode without the need of additional hole-transporting-layer. *Appl. Phys. Lett.* **100**, 013310 (2012).
